# Optimization technique for increasing resolution in computed tomography imaging

**DOI:** 10.1016/j.mex.2023.102228

**Published:** 2023-05-21

**Authors:** I.V. Grossu, O. Savencu, M. Verga, N. Verga

**Affiliations:** a Coltea Clinical Hospital, I.C. Bratianu 1, Bucuresti 030171, Romania; b “Carol Davila” University of Medicine and Pharmacy, Dionisie Lupu 37, Bucuresti 020021, Romania; c Emergency University Hospital, Splaiul Independentei, 169, Bucuresti 050098, Romania

**Keywords:** Super-resolution, Medical imaging, Computed tomography, X-Rays attenuation, Interpolation, Computed tomography compatible interpolation

## Abstract

Starting from the importance of conforming to biological reality in medicine, in this paper we propose an optimization technique for increasing resolution of computed tomography (CT) images acquired using various existing scanners. Considering a three-dimensional Hounsfield Units (HU) array, together with the corresponding spatial metadata of interest (pixel sizes and slice thickness), the procedure is based on halving each voxel along the directions of the device's Cartesian frame of reference and find those values which are both satisfying the X-Rays attenuation coefficient average requirement and minimizing the HU distance to classical interpolation points. The discussed method was tested by implementing a C# .Net 6, cross-platform library containing two algorithm flavors that could be independently applied: “Z” for doubling the number of slices, and “XY” for doubling the resolution of individual slices. This design allows also chaining (e.g. one could apply the “Z,XY,Z” sequence in order to reduce four times slice thickness). In the context of existing unavoidable limitations, the first results are suggesting the “CT compatible” interpolation technique could provide a reasonable approximation of reality. However, the main advantage comes from satisfying mass conservation, which is of high importance in medical diagnosis and treatment.

• The Hounsfield Units scale is defined as a linear transformation of the X-Rays attenuation coefficients. Thus, splitting a computed tomography voxel into two congruent volumes must satisfy the HU average requirement (the initial value must equal the average of the two output HU values).
• Existing interpolation methods (linear, spline, etc.) are not compatible with the computed tomography HU average requirement. This could also result in mass estimate anomalies with significant impact in medical diagnosis.
• The proposed “CT compatible” interpolation method is based on finding those values which are both satisfying the X-Rays attenuation coefficient average requirement and minimizing the Hounsfield Units distance to classical interpolation points.

## Graphical abstract

**Figure:**
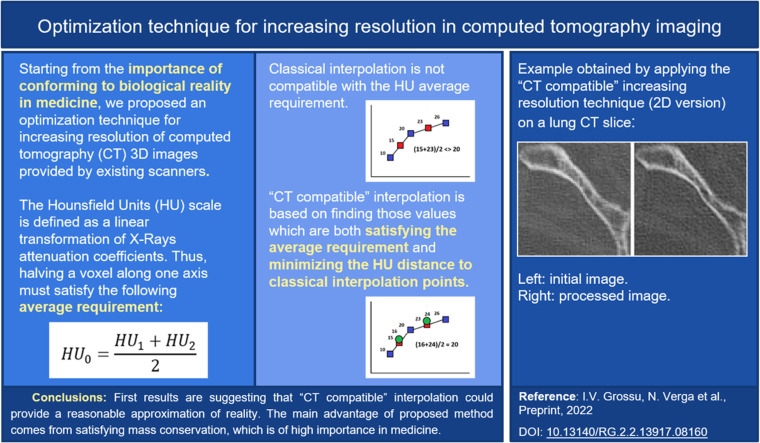

Specifications table

**Table:** 

Subject area:	Bioinformatics
More specific subject area:	Computed tomography imaging
Name of your method:	Computed tomography compatible interpolation:
Name and reference of original method:	n/a
Resource availability:	n/a

## Method details

### Introduction

The insufficient resolution of medical images (computed tomography (CT) [1], mammography [2], magnetic resonance imaging (MRI) [3], optical coherence tomography (OCT) [4] etc.) represents a significant limitation in present medicine. The physician is often confronted with lack of information when important decisions are to be taken. Waiting for future, more advanced acquisition technologies, a considerable number of recent studies were focused on developing computational methods for post-processing images provided by various existing scanners. Thus, an important attention was paid to achieving super-resolution [5], [6], [7] by employing artificial intelligence, while classical interpolation (linear [8], spline [9,10], etc.) could be used in regular resolution improvement techniques. Starting from the importance of conforming to biological reality in medicine, in this paper we propose a CT interpolation method designed in agreement with the Hounsfield Units (HU) average constraint imposed by the physics of X-Rays attenuation [1,11]. As HU and density are related [12], this also results in satisfying mass conservation, which is crucial in medical diagnosis and treatment (e.g. dosimetry [2,11], treatment of urinary stones [13], etc.).

### Method description

The HU scale [12] is defined as a linear transformation of the X-Rays attenuation coefficients. Thus, splitting a voxel in two congruent volumes must conform to the below average requirement, imposed by the laws of physics (exponential attenuation) [11]:

(1) $$HU_{0}=\frac{HU_{1}+HU_{2}}{2}$$

where HU_0_ is the initial value, while HU_1_, and HU_2_ are the HU values of the two resulting “half-voxels”.

The proposed CT resolution increment method is based on halving each voxel along the directions of the device's Cartesian frame of reference. Thus, considering a HU three-dimensional array *HU(n_x_, n_y_, n_z_)*, together with the corresponding voxel sizes metadata, an array of order *(2n_x_, 2n_y_, 2n_z_)* will be produced. As tomography images are usually represented in form of 2D slice series, it was considered more appropriate to implement two algorithm flavors, that could be independently applied: “Z” (1D), for doubling the number of slices, and “XY” (2D) for doubling the resolution of individual slices. This design allows also chaining (e.g. applying the “Z,XY,Z” sequence will result in reducing slice thickness four times).

For simplicity, the discussion will be focused on the one-dimensional “Z” flavor (“XY” being analogous). In the frame of this approach, each voxel is divided, along the *OZ axis,* into two congruent objects. The Eq. (1) shows that the HU values of resulting halves are not independent:

(2) $$\propto \;\overset{\text{def}}{=}HU_{0}-HU_{1}=>HU_{2}=HU_{0}+\propto$$

where *α* is a number to be evaluated.

One could consider the Euclidean distance, defined in the space of HU values:

(3) $$d_{ij}=\sqrt{{\left(HU_{i}-HU_{j}\right)}^{2}\;}\;$$

where *HU_i_*, and *HU_j_* denote the HU values of two random voxels (*i*, and *j*).

Finding *α* in Eq. (2) could be treated from an optimization problem perspective. Thus, the HU distance Eq. (3) was used for defining the following objective function:

(4) $$f\left(\alpha \right)=d_{1l}^{2}+\;d_{2r}^{2}={\left(HU_{1}-HU_{l}\right)}^{2}+{\left(HU_{2}-HU_{r}\right)}^{2}\;={\left(HU_{0}-\propto -HU_{l}\right)}^{2}+{\left(HU_{0}+\propto -HU_{r}\right)}^{2}$$

where *HU_l_* and *HU_r_* represent a set of “reference” values with respect to which the previous function should be minimized.

Eq. (4) is a second-degree polynomial in *α:*

(5) $$f\left(\alpha \right)=a\alpha^{2}+b\alpha +c$$

where *a, b*, and *c* are the polynomial coefficients that are immediately resulting from evaluating the parentheses in Eq. (4).

Thus, the minimum of *f* is reached at:

(6) $$\alpha_{min}=-\frac{b}{2a}=\;\frac{HU_{r}-HU_{l}}{2}$$

The reference in Eq. (6) was chosen by employing linear interpolation [8]:

(7) $$\begin{array}{ccc} HU_{l} & = & \frac{HU_{0}\left(x,y,z-1\right)+HU_{0}\left(x,y,z\right)}{2}\\ HU_{r} & = & \frac{HU_{0}\left(x,y,z+1\right)+HU_{0}\left(x,y,z\right)}{2} \end{array}$$

where *x, y,* and *z* are the HU array indices (directly related to spatial coordinates). Thus*, (x,y,z-1)*, and *(x,y,z* *+* *1)* are the coordinates of the two adjacent voxels along the OZ axis.

The minimum is attained at:

(8) $$\alpha_{min}=\;\frac{HU_{0}\left(x,y,z+1\right)-HU_{0}\left(x,y,z-1\right)}{4}$$

Eq. (8), by its linearity, is convenient from both computational point of view and propagation of errors [9]. Another option, that could be more relevant from biological perspective but more expensive in terms of computational costs, would be to choose the reference by spline interpolation [9,10].

It is also important to mention that the minimum output HU values were limited to air (*−1024*).

## Method testing

For testing the discussed method, a C# .Net 6 [14] cross-platform library was developed in agreement with SOLID principles [15]. Working with Digital Imaging and Communications in Medicine (DICOM) files is not straightforward in .Net. This is the main reason it was preferred employing the comma separated values (csv) file format that we designed for the “Hyper-Fractal Analysis” .Net application [16]. Similar with DICOM, the information is structured as a series of files (slices), each one containing the corresponding uncompressed HU two-dimensional array. One additional file is used for storing only the metadata of interest (e.g. pixel size, slice thickness, etc.).

In Table 1 is presented a comparative one-dimensional example for all linear, cubic spline, and “CT compatible” interpolation methods. The study is based on three colinear equidistant congruent segments (*AXE, BY*, and *CZ*) characterized by the corresponding HU values: HU_0A_=0, HU_0B_ *in [−50,150]*, and *HU_0C_=100*. The values resulted from splitting *BY* into two congruent segments are HU_1B_*, and* HU_2B._

**Table 1:** One-dimensional example for comparing all linear, cubic spline, and “CT compatible” interpolation methods. All values are expressed in HU.

	Linear			Cubic Spline			CT Compatible		
HU_0B_	HU_1B_	HU_2B_	Δ_B_	HU_1B_	HU_2B_	Δ_B_	HU_1B_	HU_2B_	Δ_B_
−50	−25	25	50	−43.75	6.25	31.25	−75	−25	0
−40	−20	30	45	−36.875	13.125	28.125	−65	−15	0
−30	−15	35	40	−30	20	25	−55	−5	0
−20	−10	40	35	−23.125	26.875	21.875	−45	5	0
−10	−5	45	30	−16.25	33.75	18.75	−35	15	0
0	0	50	25	−9.375	40.625	15.625	−25	25	0
10	5	55	20	−2.5	47.5	12.5	−15	35	0
20	10	60	15	4.375	54.375	9.375	−5	45	0
30	15	65	10	11.25	61.25	6.25	5	55	0
40	20	70	5	18.125	68.125	3.125	15	65	0
50	25	75	0	25	75	0	25	75	0
60	30	80	5	31.875	81.875	3.125	35	85	0
70	35	85	10	38.75	88.75	6.25	45	95	0
80	40	90	15	45.625	95.625	9.375	55	105	0
90	45	95	20	52.5	102.5	12.5	65	115	0
100	50	100	25	59.375	109.375	15.625	75	125	0
110	55	105	30	66.25	116.25	18.75	85	135	0
120	60	110	35	73.125	123.125	21.875	95	145	0
130	65	115	40	80	130	25	105	155	0
140	70	120	45	86.875	136.875	28.125	115	165	0
150	75	125	50	93.75	143.75	31.25	125	175	0

Linear interpolation [8] is straightforward: HU_1B_=(HU_0A_+ HU_0B_)/2*, and* HU_2B_=(HU_0B_+ HU_0C_)/2.

Spline interpolation [9,10] was applied on the following 3 points: (*0, HU_0A_*), (*1, HU_0B_*), and (*2, HU_0C_*), resulting in two cubic polynomials *q_1_*, and *q_2_*. Thus: HU_1B_= *q_1_(0.5), while* HU_2B_= *q_2_(1.5).*

The below measure was defined for verifying the average requirement Eq. (1):

(9) $$\Delta \;=\left|\frac{HU_{1}+HU_{2}}{2}-HU_{0}\;\right|$$

**Fig. 1:**
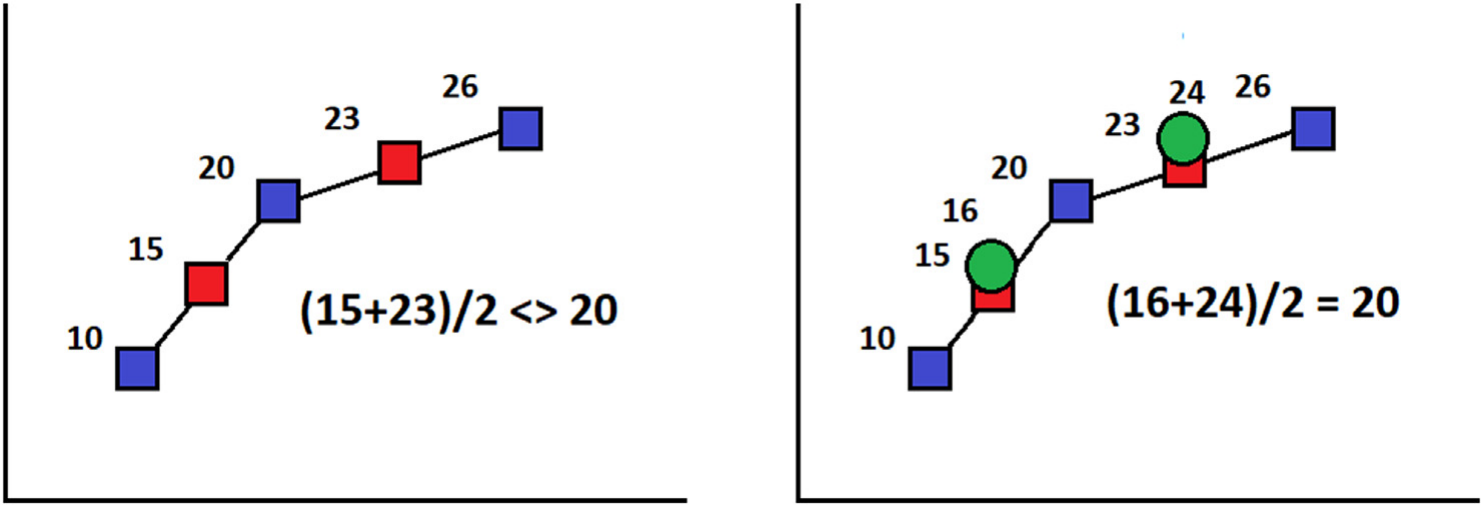
Linear vs “CT compatible” interpolation methods. Red squares: linear interpolation points. Green circles: the values which are both satisfying the average requirement and minimizing the HU distance to classical interpolation points (red squares).

As expected, Δ equals zero only for CT compatible interpolation (which is also illustrated in Fig. 1).

**Fig. 2:**
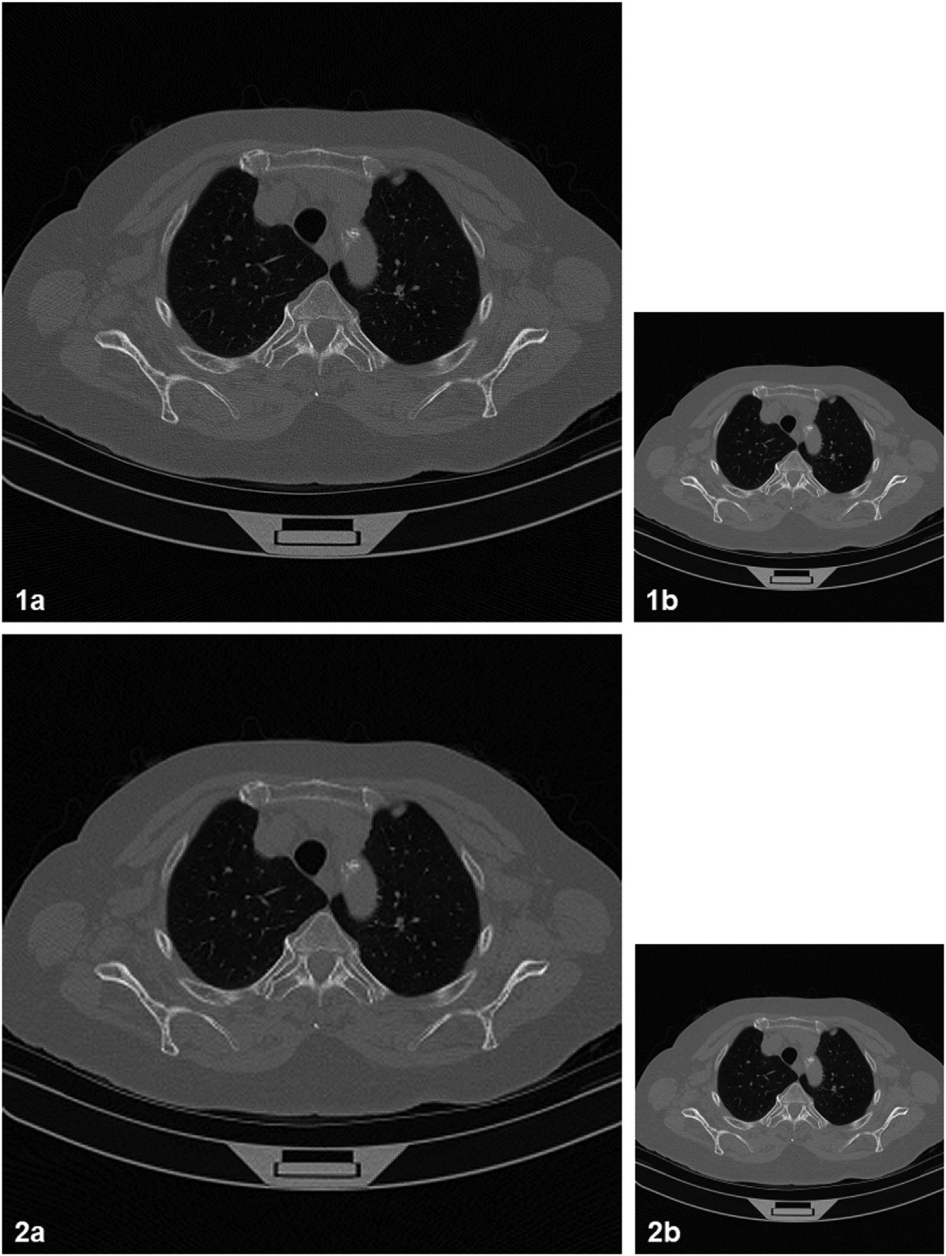
CT compatible method, “XY” (2D) algorithm applied on a lung CT slice with lowered resolution. 1a: initial CT slice: 512 × 512 voxels (0.7 × 0.7 × 1.5 mm). 1b: The image 1a after reducing its resolution to 256 × 256 voxels (1.4 × 1.4 × 1.5 mm). 2a: the result obtained by increasing the resolution of 1b (2a could be considered a reasonable approximation of 1a). 2b: The image 2a after reducing its resolution to 256 × 256 voxels (1b and 2b are identical).

The main difficulty in validating CT increasing resolution algorithms comes from the unavailability of high-resolution information to be used as reference. As a workaround, one could consider employing images with lowered resolution. This way, it is possible to compare the algorithm output with the original CT slices. In Fig. 2 is presented an example of applying the “XY” (2D) algorithm on a lung CT slice with lowered resolution: from 512 × 512 (0.7 × 0.7 × 1.5 mm) to 256 × 256 (1.4 × 1.4 × 1.5 mm) voxels. One could notice that, despite the unavoidable loss of information, the output could be considered a reasonable approximation of reality. It is also important to mention that, by lowering the resolution of both initial and processed files, identical results (csv data files) are obtained, which shows that the previously discussed average constraint (Eq. (1)) is satisfied.

**Fig. 3:**
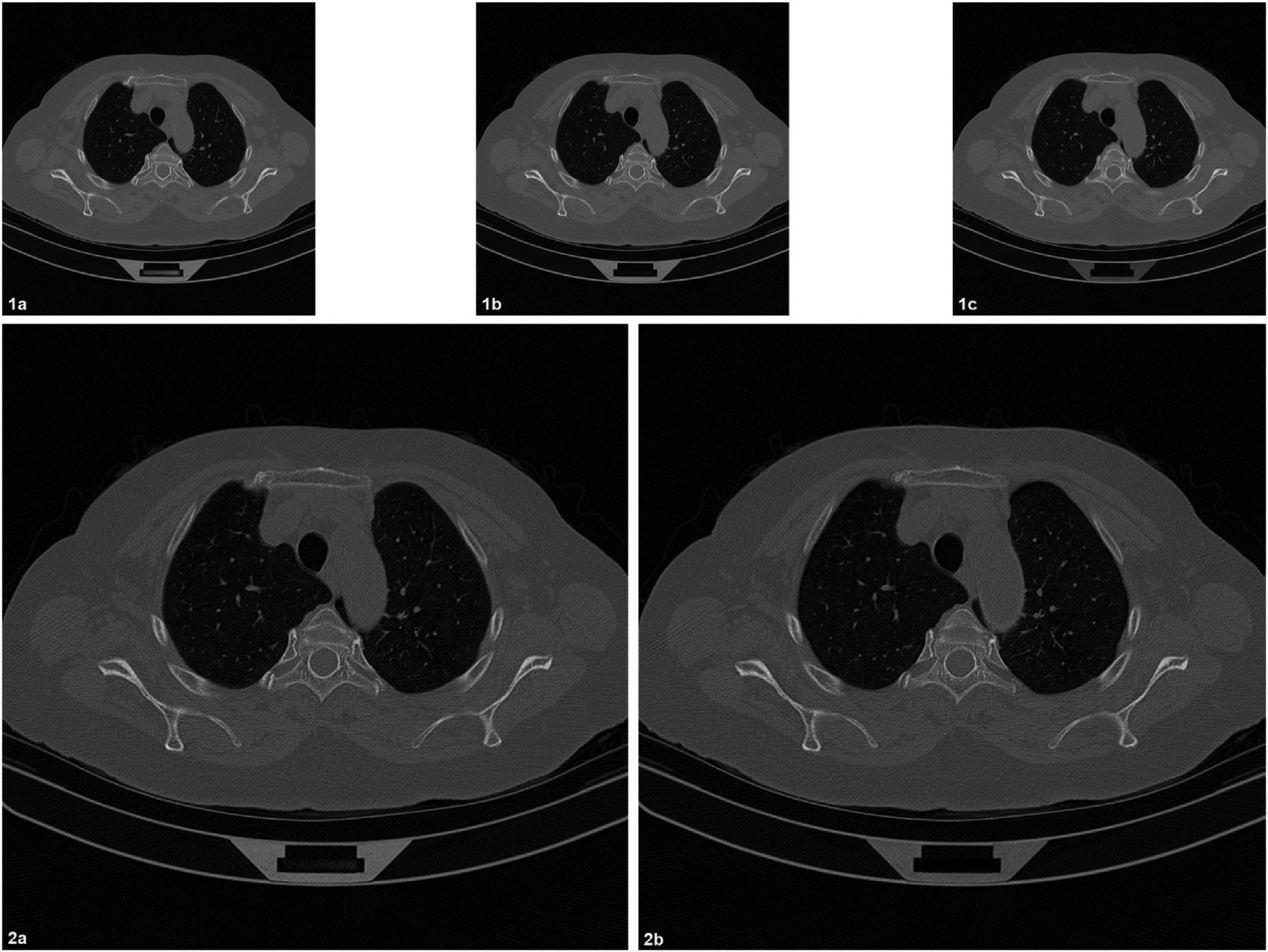
CT compatible method, “Z, XY” algorithm chain (3D) applied on a lung CT series composed by 3 adjacent slices at 512 × 512 voxels (0.7 × 0.7 × 1.5 mm). 1a, 1b, 1c: initial CT series. 2a, 2b: algorithm output: 2 slices at 1024 × 1024 voxels (0.35 × 0.35 × 0.75 mm).

In Fig. 3 is also presented the result obtained by applying the “Z, XY” algorithm chain (3D) to a set of 3 lung CT adjacent slices acquired at 512 × 512 voxels (0.7 × 0.7 mm, and slice thickness 1.5 mm). Thus, the central slice is first split (“Z”) into two new slices with half of the original thickness (0.75 mm). The resolution of each slice is after increased (“XY”) to 1024 × 1024 voxels (0.35 × 0.35 × 0.75 mm). One could notice that each output slice is most influenced by its closest neighbors from the initial series.

## Conclusions

Waiting for future, more advanced acquisition technologies and trying to conform, as much as possible, to biological reality, we proposed an optimization technique for increasing resolution of images provided by existing computed tomography scanners. The discussed method is based on halving each voxel along the directions of the device's Cartesian frame of reference and finding those values which are both satisfying the X-Rays attenuation coefficients average requirement and minimizing the HU distance to classical interpolation points. A .Net 6, cross-platform library was developed for testing purposes. It contains two algorithm flavors that could be independently applied: “Z” for doubling the number of slices, and “XY” for doubling the resolution of individual two-dimensional images. This design allows also chaining (e.g. applying the “Z,XY,Z” sequence will result in reducing slice thickness four times). In the context of existing unavoidable limitations, the first results are suggesting the “CT compatible” interpolation method could provide a reasonable approximation of reality. On the other hand, as HU and density are related, the HU average requirement is also compatible with mass conservation, which is of high importance in medical diagnosis and treatment.

## Ethics statements

The paper includes data for only one person (Olimpiada Grossu) from which the consent was obtained.

## CRediT authorship contribution statement

**I.V. Grossu:** Conceptualization, Methodology, Software, Writing – original draft. **O. Savencu:** Resources, Data curation, Validation. **M. Verga:** Conceptualization, Validation. **N. Verga:** Supervision, Conceptualization, Validation.

## Declaration of Competing Interest

The authors declare that they have no known competing financial interests or personal relationships that could have appeared to influence the work reported in this paper.

## Data Availability

The data that has been used is confidential.

## References

[1] Seeram E. (2015).

[2] Scarlat F., Scarisoreanu A., Verga N. (2013). Absorbed dose distributions using the isodensitometric method for exposures with filter employed for mammographies. Rom. Rep. Phys..

[3] Ruiz de Miras J., Navas J., Villoslada P., Esteban F.J. (2011). UJA-3DFD: a program to compute the 3D fractal dimension from MRI data. Comput. Methods Prog. Biomed..

[4] Verga N., Mirea D.A., Busca I., Poroschianu M.N., Zarma S.F., Grinisteanu L., Anica A., Gheorghe C.A., Stan C.A., Verga M., Vasilache R. (2017). Optical coherence tomography in oncological imaging. Rom. Rep. Phys..

[5] Park J. (2018). Computed tomography super-resolution using deep convolutional neural network. Phys. Med. Biol..

[6] Kitahara H., Nagatani Y., Otani H. (2022). A novel strategy to develop deep learning for image super-resolution using original ultra-high-resolution computed tomography images of lung as training dataset. Jpn. J. Radiol..

[7] F. Tatsugami et al., Improvement of spatial resolution on coronary CT angiography by using super-resolution deep learning reconstruction, academic radiology, January 2023, ISSN 1076-6332, doi:10.1016/j.acra.2022.12.044. 36681533

[8] Rajon D.A., Bolch W.E. (2003). Marching cube algorithm: review and trilinear interpolation adaptation for image-based dosimetric models. Comput. Med. Imaging Graph..

[9] Landau R.H., Paez M.J., Bordeianu C.C. (2007).

[10] Prenter P.M. (1975).

[11] Ivanov V.I. (1999).

[12] Sudhyadhom A. (2020). On the molecular relationship between Hounsfield Unit (HU), mass density, and electron density in computed tomography (CT). PLoS One.

[13] Gücük A., Üyetürk U. (2014). Usefulness of hounsfield unit and density in the assessment and treatment of urinary stones. World J. Nephrol..

[14] Whitesell R.R., Groves M.D. (2022). Pro Microservices in .NET 6.

[15] Joshi B. (2016). Beginning SOLID principles and design patterns for ASP.NET developers. Apress.

[16] Grossu I.V., Savencu O., Miron A.I., Besliu C., Verga N. (2022). Medical module for hyper-fractal analysis. Comput. Phys. Commun..

